# Cellulosic Ethanol Production Using a Dual Functional Novel Yeast

**DOI:** 10.1155/2022/7853935

**Published:** 2022-03-07

**Authors:** Z. Lewis Liu, Bruce S. Dien

**Affiliations:** BioEnergy Research Unit, National Center for Agricultural Utilization Research, USDA-ARS, Peoria, IL 61604, USA

## Abstract

Reducing the cost of cellulosic ethanol production, especially for cellulose hydrolytic enzymes, is vital to growing a sustainable and efficient cellulosic ethanol industry and bio-based economy. Using an ethanologenic yeast able to produce hydrolytic enzymes, such as *Clavispora* NRRL Y-50464, is one solution. NRRL Y-50464 is fast-growing and robust, and tolerates inhibitory compounds 2-furaldehyde (furfural) and 5-hydroxymethyl-2-furaldehyde (HMF) associated with lignocellulose-to-fuel conversion. It produces three forms of *β*-glucosidase isozymes, BGL1, BGL2, and BGL3, and ferment cellobiose as the sole carbon source. These *β*-glucosidases exhibited desirable enzyme kinetic parameters and high levels of enzyme-specific activity toward cellobiose and many oligosaccharide substrates. They tolerate the product inhibition of glucose and ethanol, and are stable to temperature and pH conditions. These characteristics are desirable for more efficient cellulosic ethanol production by simultaneous saccharification and fermentation. NRRL Y-50464 provided the highest cellulosic ethanol titers and conversion rates at lower cellulase loadings, using either pure cellulose or agricultural residues, as so far reported in the literature. This review summarizes NRRL Y-50464 performance on cellulosic ethanol production from refined cellulose, rice straw, and corn stover processed in various ways, in the presence or absence of furfural and HMF. This dual functional yeast has potential to serve as a prototype for the development of next-generation biocatalysts. Perspectives on continued strain development and process engineering improvements for more efficient cellulosic ethanol production from lignocellulosic materials are also discussed.

## 1. Introduction

Renewable cellulosic ethanol as an advanced biofuel is an attractive alternative for transportation use to reduce fossil fuel consumption and a cleaner environment. However, commercializing cellulosic ethanol production poses significant challenges. Lignocellulosic biomass pretreatment procedures, especially dilute-acid pretreatment, typically generate toxic chemicals as by-products such as 2-furaldehyde (furfural) and 5-hydroxymethyl-2-furaldehyde (HMF), which inhibit microbial growth and fermentation [1–4]. Carbohydrates imbedded in plant fibers such as cellulose and holocellulose need to be hydrolyzed to monosaccharides prior to microbial fermentation. Additional enzymes including cellulase, *β*-glucosidase, and auxiliary enzymes are required for enzymatic hydrolysis and saccharification. In a conventional simultaneous saccharification and fermentation (SSF) process for cellulosic ethanol production, cellulase hydrolyzes cellulose into oligoglucans and cellobiose, and additional *β*-glucosidase converts cellobiose into glucose for yeast fermentation. Beta-glucosidase (*β*-D-glucoside, glucohydrolase, EC 3.2.1.21) hydrolyzes nonreducing *β*-D-glucosyl residues from glycosides and *β*-linked oligosaccharides, releasing glucose. Enzyme costs for cellulosic ethanol are approximately 10 times greater than conventional starch-based fermentations using amylases [5, 6]. As a critical enzyme for hydrolyzing cellulose to glucose, *β*-glucosidase has drawn considerable attention in recent years within the context of cellulosic ethanol production [7]. Overcoming toxic compounds, reducing enzyme cost, and improving efficiency of cellulosic ethanol fermentation are among the significant challenges that need to be solved for economic production of renewable cellulosic ethanol. Significant progress has been made in the past decades, yet challenges remain for sustainable and economic production of cellulosic ethanol from lignocellulosic materials in industrial applications.

Since most ethanol-fermenting microbes do not synthesize cellulose hydrolytic enzymes, engineering efforts have been made to enable ethanologenic yeast to produce *β*-glucosidase [8–15]. However, these strains have insufficient *β*-glucosidase activity for efficient cellulosic ethanol production by SSF. Recent studies have found a naturally occurring *Clavispora* yeast, which was isolated from sweet sorghum, that can produce *β*-glucosidase and ferment cellobiose to ethanol. A thermal-tolerant and furfural- and HMF-resistant strain was generated from its progenitor wild-type strain, namely, *Clavispora* NRRL Y-50464 through adaptive laboratory evolution [16]. Strain NRRL Y-50464 has the potential to lower the cost of producing cellulosic ethanol because it is a fast-growing yeast, produces sufficient native *β*-glucosidase enzyme activity for SSF, and can utilize cellobiose as a sole carbon source to produce ethanol. Its cellulosic conversion performance has been investigated by scientists from the U.S.A, China, and India using a wide variety of substrates and feedstocks. NRRL Y-50464 harbors a *β*-glucosidase family with at least three members of BGL1, BGL2, and BGL3. This review summarizes current knowledge on *β*-glucosidase enzymes produced by NRRL Y-50464 and their cellulosic ethanol production from refined cellulose and agricultural residues. Perspectives on future strain development and improved process engineering for increased ethanol productivity are also proposed for continued investigations.

The adapted strain of *Clavispora* NRRL Y-50464 is a fast-growing yeast with a growth rate that surpasses *Saccharomyces cerevisiae* on glucose. It can utilize cellobiose as sole source of carbon and produces sufficient native *β*-glucosidase activity for ethanol production by SSF [17, 18]. When challenged with 15 mM each of furfural and HMF, a culture of NRRL Y-50464 quickly overcame the chemical stress after a brief lag phase and completed the ethanol fermentation within 32 h. It converted furfural into nonharmful hydroxymethylfuran (furan methanol or FM) in less than 12 h and HMF into 2,5-bishydroxymethylfuran (furan-2,5-dimethanol or FDM) in less than 24 h, while producing ethanol [16, 19]. In a study growing yeast in corn stover hydrolysates, NRRL Y-50464 showed more resistant to acetic acid than *S. cerevisiae* DQ1 [20]. However, its tolerant characteristics against acetic acid have not been quantitatively validated yet. Strain NRRL Y-50464 grows vigorously at 37°C, which is a suitable temperature for ethanol production using SSF.

## 2. Basic Assessment

### 2.1. Performance on Pure Cellulose

Strain NRRL Y-50464 fermented purified cellulose substrate Avicel or SigmaCell™ fermented equally well in SSFs under the following conditions: a freshly prepared overnight culture of NRRL Y-50464 cells was added at a ratio of 60 mg/mL wet weight to make a total volume of 25 mL for fermentation. A commercial cellulase (Celluclast 1.5 L) was added at a concentration of 15.3 filter paper unit (FPU) per Gram of cellulose material. No additional *β*-glucosidase enzyme was added. The fermentation was carried out in 100-mL Nalgene bottles vented using a 23-gauge needle with agitation at 250 rpm at 37°C. Ethanol production increased proportionally with solids loading of cellulose increased from 2 to 14%. The maximum ethanol titer of 47 g/L was achieved with 14% cellulose loading within 120 h [21]. In general, the ethanol fermentation rate decreased with increased concentrations of cellulose. The fermentation rate was maximum at 8 h for each cellulose loading and dropped sharply thereafter. From 48 h to 72 h, the conversion rate had a similar slow trend for most concentrations. Most of the ethanol was produced within 72 h with a typical ethanol production efficiency above 60%. After 72 h, the rate of fermentation slowed considerably with limited ethanol production [21]. Therefore, ethanol titer at 72 h is the optimal time to evaluate strain fermentation performance. At 72 h, an ethanol titer of 40.44 g/L was observed from a solids loading of 14% cellulose, equating to a conversion efficiency of 56.6%.

Its performance for SSF of refined cellulose without the addition of *β*-glucosidase is superior to that of most engineered strains (Table 1). For example, a genetically engineered *S. cerevisiae* strain produced 24 g/L ethanol after 72 h from 8% refined cellulose without supplementing *β*-glucosidase activity [13]. Strain NRRL Y-50464 produced 26.54 g/L ethanol from 8% pure cellulose at 72 h, which equals an efficiency of 65%. It took 96 h for the *S. cerevisiae* strain to reach this titer. An engineered bacterial strain produced approximately 25 g/L ethanol from pure cellulose at 72 h and reached a higher titer of 38 g/L in 146 h [27]. NRRL Y-50464 produced a significantly higher titer of 47.74 g/L by 120 h from a pure cellulose [21]. Other reported engineered strains showed much lower conversion efficiencies and ethanol titers [23]. Strain NRRL Y-50464 thus far demonstrates the best product yield and rate for pure cellulose-to-ethanol conversion by SSF without supplemental *β*-glucosidase.

### 2.2. Pure Cellulose versus Corn Stover

Cellulosic ethanol production is commonly investigated using pure cellulose or crop biomass feedstocks. In order to evaluate strain performance on available cellulose content, it is important to compare metrics of ethanol conversion from refined cellulose and lignocellulose on an equal basis of the cellulose content. Standard corn stover pretreated by DOE-NREL (Department of Energy-National Renewable Energy Laboratory) protocol contains 37.84% cellulose content on a dry weight basis, which is consistent with the value listed in the DOE Biomass Feedstock Composition and Property Database (http://www.afdc.energy.gov/biomass/progs/search1.cgi). Therefore, a solids loading range of 5 to 37% standard DOE-NREL-pretreated corn stover contains 1.89 to 14.0% cellulose [21].

Under the same SSF culture conditions, ethanol conversion efficiencies for corn stover are significantly lower than that for pure cellulose. For example, an ethanol titer of 20 g/L from 6% pure cellulose is achieved within 48 h. In contrast, it took 120 h with an equivalent cellulose-based loading of corn stover at 15% to reach this level. Also, refined cellulose concentrations of 8% and 10% often resulted in much higher ethanol titers than equivalent cellulose loadings using the pretreated corn stover at 20 and 25% total biomass solids loadings. Overall, the conversion efficiencies using corn stover are only 50–70% compared to that using pure cellulose [21]. Therefore, there is significant potential to improve ethanol production from corn stover. In this challenging area, lignin stands out among the numerous factors as a major barrier to realizing more complete cellulose utilization from corn stover.

### 2.3. Standard Corn Stover versus Delignified Corn Stover

Detailed experimental procedures are available elsewhere [21]. Briefly, solid cellulose materials of conventional dilute-acid-pretreated corn stover obtained from DOE-NREL were treated with 6 N NaOH to adjust a pH value at 5.5. The solids were then washed with four volumes of Milli-Q water to remove residual salt. The materials were dried at 60°C overnight for the measurement of dry weight of cellulose. The same source of conventional pretreated corn stover solids was treated by an additional delignification procedure using hydrogen peroxide as previously described [28]. The treated biomass was incubated for 16 h at room temperature with gentle mixing. The slurry was then filtered off and washed with water until the filtrate is clear. The solids were adjusted to pH 5.5 and dried for weight measurement. Fermentations were carried out using water insoluble solid (WIS) contents of 15, 20, and 25% (dry weight) solids loading in a final concentration without glucose. The fermentation was conducted in a total volume of 50 mL in 100-mL Nalgene bottles with a 23-gauge needle venting as described above. Enzyme addition and cell inoculation of NRRL Y-50464 were the same as described above. The SSF was carried out in a 37°C incubator with agitation at 250 rpm. For comparison studies, all strains were treated in the same way under the same conditions and details available in the original reports.

Fermentation of delignified corn stover showed significantly higher ethanol titer, conversion rate, and efficiency than that for DOE-NREL-pretreated corn stover (e.g., lignin intact) under the same SSF culture conditions. Notable, the performance using delignified biomass was similar to that using pure cellulose. Ethanol conversion from the delignified corn stover cellulose was completed in 72 h for solids loading levels of 15, 20, and 25%. In contrast, it took over 120 h to complete the fermentation using DOE-NREL dilute-acid-pretreated corn stover [21]. Ethanol titers of delignified corn stover increased from 30 to 40% and the conversion efficiencies increased from 40 to 60% compared with the DOE-NREL-pretreated corn stover. The conversion rate was significantly higher for delignified corn stover than that for the DOE-NREL-pretreated corn stover at all solids loadings with the highest conversion rate (0.0531 g L^−1^h^−1^) at 15% solids loading. For more concentrated slurries, the rates decreased with increased solids loadings, though still similar to those using high solids pure cellulose [21].

This demonstrates that lignin is the dominant factor impeding corn stover-to-ethanol conversion. Cellulose morphology has been observed to influence the enzymatic digestion of cellulose in pretreated corn stover [29]. Delignification removed nonproductive adsorption sites that bind cellulases while increasing the accessibility of the cellulose fiber, thus improving its digestibility [30, 31]. Since the ethanol titer from delignified corn stover was still lower than that from equivalent pure cellulose at higher solids loading levels, there are also other interfering factors. However, increasing the cellulase dose did not improve the fermentation efficiency, which suggests cellulase was not limiting the rate or yield (Liu, unpublished data). More efficient biomass pretreatment strategies are expected to provide more effective downstream processing for enzyme hydrolysis and ethanol production [32]. At very high solids concentrations, the wetted biomass forms a viscous slurry, which is not well mixed in a flask or conventional bioreactor system. Poor mixing also reduces the efficiency of ethanol fermentation. The role of process engineering to improve SSFs is discussed below.

## 3. Ethanol Production from Agricultural Materials

### 3.1. Industrial Processed Corncob Residue

Corncobs are used commercially for xylose production. After xylose extraction, the residual cake, which contains greater than 50% cellulose, can be used for cellulosic ethanol production [33]. However, the high levels of toxic furfural present in the residual corncob cake combined with the high cost of detoxification and slow fermentation rates hinder its use for ethanol production [14, 33]. In this case, strain Y-50463 is a suitable fermentation host because it is robust to furfural, has a fast conversion rate, and produces its own *β*-glucosidase.

Strain NRRL Y-50464 produced 26.6 g/L ethanol from SSF using 20% solids of industrial processed corncob residue in 5 days without supplementing with *β*-glucosidase [16]. In contrast, a control fermentation inoculated with *S. cerevisiae* failed to ferment a significant amount of cellobiose and only produced a trace of ethanol, likely from pre-existing glucose. The efficiency of ethanol conversion from the corncob residue decreased when solids loadings were increased from 15 to 35% in SSF using NRRL Y-50464. While the maximum yield efficiency (55%) was realized at 15% solids loading, the highest ethanol titer was obtained at 25% solids loading [16]. A similar conversion efficiency (53%) was previously reported for 15% solids loading of corncobs [34]. In SSF with added *β*-glucosidase, both ethanol production and conversion efficiency were lower than without the addition of the enzyme at 15 and 25% solids loadings. Adding extra *β*-glucosidase in fact did not show any benefit for ethanol production via SSF in numerous repeated fermentations [16]. A previous report also observed a slightly higher ethanol titer without added *β*-glucosidase than with [13]. After 17-h incubation, NRRL Y-50464 produced 1.20 U/mg/ml specific *β*-glucosidase activity [16]. This high level of early expression is sufficient enzyme activity for cellobiose hydrolysis and ethanol fermentation in SSF. Whether overdosing *β*-glucosidase interferes with cellobiose hydrolysis and fermentation in SSF remains unclear. Using a 2-L bioreactor for SSF, NRRL Y-50464 produced 23 g/L ethanol in 120 h without the addition of *β*-glucosidase [16].

Another more reliable method to calculate conversion efficiency is to measure total glucans prior to and after SSF [35]. Using this analysis method, NRRL Y-50464 showed the highest glucose consumption of 77.36 and 53.98 g/L for 25% and 15% solids loading, respectively [16]. Naturally, ethanol production also significantly increased. As expected, the higher conversion efficiency (93% of theoretical) occurred at 15% solids loading. The efficiencies decreased significantly at 20 and 25% solids loadings. Therefore, much of the glucan was not converted to glucose at higher solids concentrations. This result suggests that better pretreatment methods are needed to release insoluble sugars for an improved ethanol yield [36].

### 3.2. DOE-NREL-Pretreated Corn Stover

**Figure 1:**
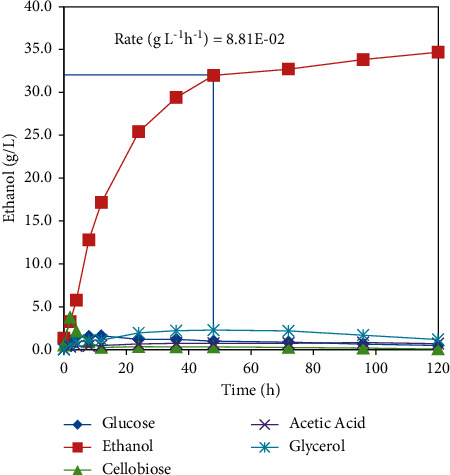
Cellulosic ethanol production using *Clavispora* NRRL Y-50464 from standard DOE-NREL-pretreated corn stover with a 20% solids loading (equivalent to 7.57% cellulose) by simultaneous saccharification and fermentation using 2-L bioreactors. Values of ethanol production are means of three replications with a standard deviation of 0.05 g/L at 48 h and ranged from 0.02 to 0.24 g/L for all time points [21].

Using strain NRRL Y-50464, DOE-NREL-pretreated corn stover at 20% solids loading yielded an ethanol titer of 34.7 g/L for a 120 h SSF conducted using a bioreactor [21]. It produced 32 g/L ethanol within 48 h at a linear rate of 0.088 g/L/h (Figure 1), which suggests the run time can be shortened. In contrast, when the SSF was conducted in a bottle culture, corn stover at the same loading produced less than 15 g/L ethanol in 48 h. Therefore, running in a bioreactor doubled the yield of ethanol and significantly improved the conversion rate compared to the bottle SSF. This comparison demonstrates the importance of a good mixing [21].

Conventional bottle fermentations using a shaker or magnetic stirring bar for mixing are commonly used for SSF. While convenient, the above example suggests that this setup may lead to subpar yields when combined with high solids. Even though the mechanically stirred bioreactor provides a better agitation, it is still not the optimal design for cellulosic ethanol production since it is designed for liquid cultures. In this case, the stirrers are typically located near the bottom of the vessel and provide limited mixing for high solid slurries. For high solids loadings of cellulosic materials, a different mixing blade mechanism with an all-around moving motion is expected to further improve the efficiency of cellulosic ethanol production at both laboratory and pilot scales.

### 3.3. Conventional Corn Stover

Performances of strain NRRL Y-50464 and *S. cerevisiae* DQ1 on conventional corn stover were compared using the fermentation of corn stover hydrolysate (CSH) by separate hydrolysis or SSF processes. For detailed methods and specific procedures, reader is referred to the original report [20]. Under both conditions, strain NRRL Y-50464 exhibited superior capability for cellobiose conversion and ethanol production over a strain of *S. cerevisiae* DQ1 [20, 37]. The ethanol titer for the NRRL Y-50464 fermentation was 38 g/L using enzyme-hydrolyzed conventional corn stover at 35% solids loading (w/w) [16]. In a different study using 25, 30, and 35% of CSH containing 13.4, 14.1, and 16.5 g/L of cellobiose, respectively, NRRL Y-50464 grew faster than *S. cerevisiae* DQ1 [20]. Strain NRRL Y-50464 produced significantly higher levels of ethanol at all solids loading levels. The ethanol production increased with the increased solids loading levels at 26 g/L for 25% CSH, and the highest at 38 g/L for 35% CSH. The increased portion of ethanol production was attributed to the cellobiose in the CSH since strain NRRL Y-50464 can directly convert cellobiose into ethanol. The 35% solids CSH contained 79.6 g/L glucose, 13.2 g/L xylose, and 16.5 g/L cellobiose. While both strains are unable to utilize xylose, additional cellobiose benefited additional ethanol production by NRRL Y-50464 [16, 20]. SSF fermentations were equally effective with ethanol titers of 37.7 g/L and 38.1 g/L with and without added *β*-glucosidase enzyme, respectively (Table 1). When *β*-glucosidase was not added to the fermentation, the ethanol titer equated to a conversion efficiency of 55.5% [20]. Slightly lower ethanol titers in SSF were commonly observed previously [13]. Whether the extra *β*-glucosidase interferes with ethanol fermentation is currently unclear.

It is notable that the SSF using conventional pretreated corn stover required only 5 mg protein/g glucan, equivalent to 6.7 FPU/g glucan, which is the lowest cellulase loading reported in literature (Table 1). The reduction in cellulase is expected to yield significant savings. Another factor in achieving a higher ethanol titer was the use of a 5-L bioreactor equipped with a helical stirring apparatus [20, 36, 38]. Unlike traditional Rushton or marine impellers, the helical stirrer is designed to mix the cellulose slurry throughout the entire vessel in a uniform fashion and is, therefore, well suited for use in cellulosic fermentations.

### 3.4. Rice Straw

Ethanol fermentation by strain NRRL Y-50464 was evaluated on rice straw after different pretreatment procedures using mild-alkali, dilute-acid, or deep eutectic solvents. Ethanol production from mild-alkali-pretreated rice straw SSFs was significantly higher than those from dilute-acid-pretreated rice straw SSFs at 10, 15, and 20% solids loadings [24]. For dilute-acid-pretreated rice straw, NRRL Y-50464 produced 16.8 g/L ethanol after 120 h from 15% solids loading. Notably, this is higher than most reported ethanol titers using dilute-acid-pretreated rice straw with a wide variety of microbes, including *S. cerevisiae,* which only produced 10.2–12.3 g/L ethanol [39]. However, ethanol yields for mild-alkali versus the dilute-acid-pretreated rice straw were much better. Ethanol production increased to 25 g/L at a uniform rate within 36 h on mild-alkali-pretreated rice straw. Its conversion efficiency on mild-alkali-pretreated rice straw was 79.2, 64.0, and 45.4% from 10, 15, and 20% solids loadings, respectively, significantly higher than those observed from dilute-acid-pretreated rice straw sugars. Therefore, mild-alkali is preferred to dilute-acid for pretreating rice straw for ethanol conversion by SSF [24].

To further increase conversion efficiency, advanced pretreatments using a set of deep eutectic solvents were investigated, which are comprised of biodegradable and eco-friendly green solvents [40]. Beyond disrupting crystalline cellulose fibers, the green solvents also extract lignin from rice straw, all of which significantly improved fermentation efficiency [26]. Strain NRRL Y-50464 tolerated selective green solvents, including choline chloride/glycerol (CC-GLY), choline chloride/1,2-propane diol (CC-PD), and choline chloride/ethylene (CC-EG), as evidenced by successful fermentation of sugars generated from the deep eutectic solvent-pretreated rice straw. With most of these pretreatments, ethanol production achieved 19.7 g/L after 24 h. An ethanol titer of 36.7 g/L was obtained within 36 h from the hydrolysate of deep eutectic solvent-pretreated rice straw at 20% solids, equating to an ethanol conversion efficiency of 89% [25].

### 3.5. Summary of Cellulosic Conversion Performance

It is difficult to compare the performances of various microbes reported for cellulosic ethanol production because of the variability in biomass contents and methods. For refined cellulose SSFs, most strains seem to produce similar ethanol titers. However, strain NRRL Y-50464 demonstrated a higher conversion efficiency with a record titer of 40.44 g/L from 14% cellulose within 72 h, and a maximum titer of 48 g/L within 120 h (Table 1). It showed about a 10% increase in titer at 72 h from processed industrial corncob residue. NRRL Y-50464 is a desirable yeast to use for corncob-to-ethanol conversion by SSF because it tolerates furfural presented in the substrate and its better conversion efficiency.

Yeast strain NRRL Y-50464 demonstrated exceptional ethanol productivities and titers for rice straw fermentations. The mild-alkali pretreatment appeared superior to the acid-treated rice straw, in part because the former removed a significant amount of lignin [25]. Evidence of lignin-associated inhibition was also demonstrated in comparative studies of delignified versus un-delignified corn stover [21]. More efficient pretreatment methods are expected to further improve cellulosic ethanol production potentials. Ethanol production of 32 g/L from DOE-NREL-pretreated corn stover was achieved within 48 h using a 2-L bioreactor via SSF, with a conversion rate at 0.0881 g L^−1^h^−1^ using DOE-NRRL Y-50464. Applying a 5-L bioreactor with a helical stirring apparatus, this strain produced an ethanol titer of 38.1 g/L from conventional corn stover by SSF, using a very low amount of cellulase (5 mg protein/g glucan or 6.7 FPU/g glucan) (Table 1). This was close to the ethanol titer observed from pure cellulose; however, it took 96 h rather than 72 h to reach this level. The conversion efficiency from the conventional corn stover was also lower than that from DOE-NREL-pretreated corn stover. It was likely variables of the pretreatment procedure of the conventional corn stover may compromise its efficiency of ethanol conversion. Overall, the fast conversion rate of NRRL Y-50464 was exceptional regardless of the cellulosic materials tested.

## 4. Evidence of *β*-Glucosidase Production

### 4.1. Expression

When grown on a mixture of glucose and cellobiose, the *β*-glucosidase activity was induced once glucose was exhausted [17, 41]. This indicated that *β*-glucosidase induction is cellobiose-dependent. In cellobiose cultures, maximum *β*-glucosidase activity of BGL1 occurred at 18 h. The rapid induction of protein expression is consistent with its fast rate of fermentation [17]. Similarly, gene expression of *BGL3* was also observed to be induced by cellobiose quickly reaching its highest mRNA abundance within 20 h. In contrast, the observed mRNA abundance for *BGL2* did not show a significant increase although its protein demonstrated a significantly higher level of *β*-glucosidase activity and the strain with the cloned gene showed a fast growth rate on cellobiose [41]. Since both transformants contain the same tightly regulated *AOX1* promoter, such results were unexpected. Whether its gene expression was affected by post-translation regulation is not clear, and its DNA-protein interactions are currently unknown.

### 4.2. Isolation and Identification

Beta-glucosidase activities from various cellular fractions of strain NRRL Y-50464 were evaluated in parallel with samples from closely related *β*-glucosidase producing yeast strains. Cellobiose-induced *β*-glucosidase activity for NRRL Y-50464 appeared to be associated with broken cells and spheroplasts, and relatively low activities were observed in the supernatants [17]. Compared to a closely related strain *C. lusitaniae* NRRL Y-5394, NRRL Y-50464 produced higher levels of *β*-glucosidase activity from all the tested fractions. Another yeast *Candida wickerhamii* NRRL Y-2563 [42], which is well known as a producer of *β*-glucosidase, showed the highest levels of enzyme activity. However, it grew slower on cellobiose and was a poor ethanol fermenter, which produced less than half of the ethanol from cellobiose compared with that of NRRL Y-50464 [17]. Currently, only three forms of *β*-glucosidase were characterized. Since the *β*-glucosidase activity was observed in all fractions of the cell extracts, additional forms of the enzyme may exist and remain to be recovered.

**Figure 2:**
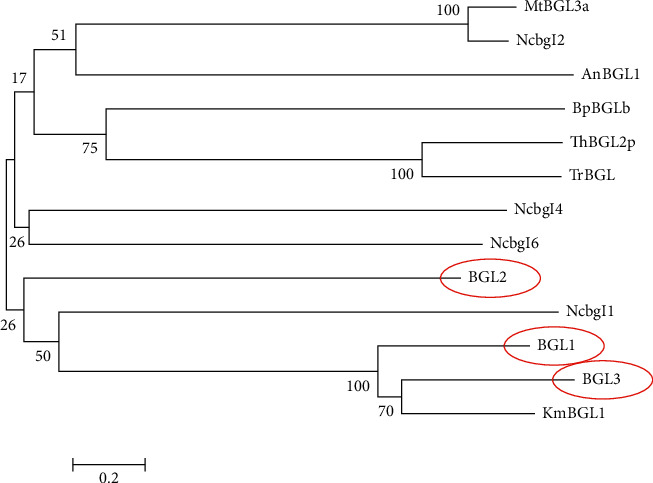
A phylogenetic tree derived from amino acid sequence analysis showing relationships of three forms of *β*-glucosidase from *Clavispora* NRRL Y-50464 (BGL1, BGL2, and BGL3) with other microbial BGLs from *Aspergillus Niger* (AnBGL1), *Trichoderma harzianum* (ThBGL2), *Trichoderma reesei* (TrBGL), *Kluyveromyces marxianus* (KmBGL1), *Myceliophthora thermophila* (MtBGL3a), *Neurospora crassa* (Ncbgl1, Ncbgl2, Ncbgl4, and Ncbgl6), and *Bacillus polymyxa* (BpBGLb) [41].

The first BGL1 was identified using 442 amino acid residues obtained from MALDI-TOF and TOF/TOF tandem MS/MS analysis. The amino acid sequence matched a hypothetical protein of CLUG_01181 from *C. lusitaniae* ATCC 42720 based on computation annotation. BGL1 was conformed to function as *β*-glucosidase by direct enzyme assay [17]. It was associated with glycoside hydrolase (GH) family 3. A catalytic nucleophile in the N-terminal domain and a proton donor in the C-terminal domain were found to be located at Asp225, Asp224, and Asp225; and Glu449, Glu458, and Glu459 for BGL1, BGL2, and BGL3 from NRRL Y-50464, respectively [17, 41, 43]. Amino acid sequences of these BGLs were distinct from other known *β*-glucosidases including *Candida tenuis, Debaryomyces hansenii, Meyerozyma guilliermondii, Scheffersomyces stipitis, Schwanniomyces etchellsii*, and *Spathaspora passalidarum* [17, 41]. A phylogenetic analysis of 13 microbial BGLs showed a close relatedness of BGL1, BGL2, and BGL3 from strain NRRL Y-50464 [41]. These results suggested there is a BGL family with at least three members in NRRL Y-50464. They were clustered with a *β*-glucosidase from another yeast species *Kluyveromyces marxianus* (Figure 2).

### 4.3. Characterization of BGLs from NRRL Y-50464

Proteins of the three BGLs from NRRL Y-50464 have a similar structure with a length ranging from 804 to 844 of amino acid residues. Molecular weight is 93.3, 88.3, and 92.5 Kda for BGL1, BGL2, and BGL3, respectively (Table 2) [17, 41].

The highest specific activity for BGL1 was observed at pH 6 (Table 2). BGL2 tolerated lower pH showing similar levels of enzyme activity at pH 4 and pH 5. BGL3 was less sensitive to pH and had similar activities from pH 4 to 6 with the maximum activity at pH 5. The optimal temperature for the highest specific enzyme activity is 45, 50, and 55°C for BGL1, BGL2, and BGL3, respectively (Table 2). BGL3 is tolerable to higher temperature and its activity remained relatively stable from 60 to 70°C [41]. Collectively, such a wide range of optimal temperature and pH performance from the three BGLs is advantageous for growing the yeast under variable fermentation conditions.

Using the optimal temperature and pH conditions, enzymatic kinetic parameters of these *β*-glucosidases were determined. All three enzymes, BGL1, BGL2, and BGL3, demonstrated significantly higher levels of substrate affinity toward p-nitrophenyl-*β*-D-glucopyranoside (pNPG) with a *K*_*m*_ ranging from 0.08 to 0.35 mM compared to Novo188 with a *K*_*m*_ of 0.448 mM (Table 3) [17, 41]. Both BGL1 and BGL2 have a superior reaction rate with significantly higher maximum enzyme velocities than Novo188, a commercial source of *β*-glucosidase.

Product inhibition of cellulases such as glucose inhibition is a major concern for cellulosic ethanol processes because it reduces hydrolysis rates and constrains the final ethanol titers. Naturally, the enzymes need to tolerate ethanol, and genetic engineering efforts have been made to increase the ethanol tolerance of *β*-glucosidase [44]. Since most *β*-glucosidases are highly sensitive to glucose inhibition, securing glucose tolerant *β*-glucosidases has become a major challenge for efficient cellulosic ethanol production [45, 46]. All the three *β*-glucosidases from NRRL Y-50464 demonstrated high levels of tolerance to glucose, and BGL2 was the least inhibited by glucose with a *K*_*i*_ of 61.97 mM (Table 3) [17, 41]. In contrast, Novo188, a commercial source of *β*-glucosidase, was more sensitive to glucose inhibition with a *K*_*i*_ of 0.735 mM. Since Novo188 is a mixture of enzymes, whether the sensitive response to glucose inhibition is affected by its compromised purity is not clear.

In addition to tolerance to glucose inhibition, BGL2 is highly resistant to product inhibition caused by ethanol [41]. Its specific enzyme activity was enhanced at lower concentrations of ethanol from 4 to 12%, which is an enhanced feature since reported cellulosic ethanol titers are typically less than 10%. However, BGL3 was highly sensitive to ethanol although it was tolerant to glucose at the extremely high concentrations up to 1000 mM [41]. Apparently, functional mechanisms of these enzymes were different in response to varied environmental conditions.

### 4.4. Substrate Specificity

Hydrolysis of cellulose releases a mixture of diversified oligosaccharides. In addition to assay using the chromogenic substrate pNPG, specific activity of BGL2 and BGL3 was evaluated toward 14 purified oligosaccharides [41]. These substrates included cellotriose, cellotetraose, cellopentose, laminaribiose, laminaritriose, laminaritetraose, laminaripentaose, laminarin, lactose, lichenan, gentiobiose, salicin, and sophorose. The hydrolytic activity of BGL2 was higher toward cellobiose with variable activities at moderate levels toward all other substrates examined [41]. On the other hand, BGL3 displayed a stronger hydrolytic activity toward most oligosaccharides when compared to cellobiose. However, the overall specific activity of BGL3 to all substrates tested, including cellobiose, was lower than that of BGL2.

## 5. Perspectives

Beta-glucosidase as an important component of cellulase is required for end-hydrolysis of cellulose-to-ethanol production by SSF. Reducing enzyme costs remains a significant challenge for commercial production of advanced biofuels. Microbes that natively produce hydrolytic enzymes typically are not homo-ethanol fermenters. The fast-growing and inhibitor robust yeast strain NRRL Y-50464 is rare in its ability to produce *β*-glucosidase and ferment cellobiose, as well as glucose, to ethanol. The yeast is also able to achieve higher ethanol titers from refined cellulose with a decent productivity. Its ethanol production properties on agricultural materials, including industrial by-product corncob residue, rice straw, DOE-NREL-pretreated corn stover, and conventional corn stover, are exceptional compared to other reported yeast strains in terms of efficiency and economics. Most significantly, the ethanol conversion rate of NRRL Y-50464 is outstanding among other available *β*-glucosidase producing strains. Identification of a *β*-glucosidase family containing at least three members of BGL1, BGL2, and BGL3 with desirable enzymatic characteristics further supports its dual function of enzyme hydrolytic functions in addition to ethanol production capability. While fast-growing and robustness of a microbial strain are desirable characteristics for industrial applications, care should be taken to prevent potential contaminations of unintended applications since NRRL Y-50464 grows faster than most yeast strains including *S. cerevisia*e. It needs to point out that the current ethanol titer by NRRL Y-50464 is still low and not ready for industrial applications. A significant strain improvement is needed to further increase its ethanol conversion capability.

NRRL Y-50464 can utilize xylose but produces xylitol rather than ethanol. This is a major defect of this strain for cellulosic ethanol production since lignocellulosic biomass contains a large portion of xylose. It is an important area, which needs to be improved in order to take a full advantage of this strain for cellulosic ethanol production using hydrolysates. Genetic engineering to redirect xylose-to-ethanol conversion pathway would significantly enhance its utilization of mixed C-5 and C-6 sugars, such as accomplished in *S. cerevisiae*. Unlike well-characterized *S. cerevisiae*, the genomic background of *Clavispora* NRRL Y-50464 is less known. Since the detoxification mechanisms of NRRL Y-50464 are similar to those observed in *S. cerevisiae*, it may not be a far-reachable goal to enable a xylose-to-ethanol pathway in NRRL Y-50464 with appropriate modifications.

**Figure 3:**
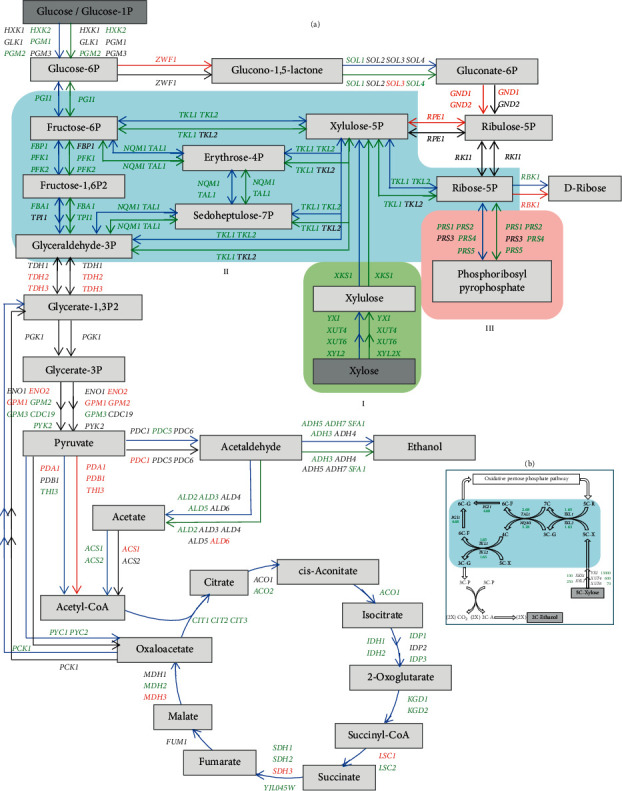
A schematic illustration of significant gene expression changes for the genetically engineered *Saccharomyces cerevisiae* NRRL Y-50463 compared with its parental wild-type industrial strain NRRL Y-12632 for endogenous genes involved in glycolysis, pentose phosphate pathway, and TCA cycle at 24 h using xylose as the sole source of carbon when glucose was depleted. The arrows on the left and the top from the parallel lines represent aerobic growth condition and those on the right side or at the bottom represent oxygen-limited fermentation condition. The blue- or green-colored arrows indicate significantly greater gene expression for aerobic and oxygen-limited conditions, respectively. The arrows in red indicate repressed expression, and the arrows in black indicate gene expression at normal or nearly normal levels. Elements of the signature expression for strain NRRL Y-50463 were boxed in various colors and labeled I, II, and III (A). An illustration of xylose transformation and metabolism through the nonoxidative pentose phosphate pathway for the genetically engineered industrial yeast *Saccharomyces cerevisiae* NRRL Y-50463. 2C-A stands for acetaldehyde; 3C-G, glyceraldehydes 3-phosphate; 3C-P, pyruvate; 4C, erythrose 4-phosphate; 5C-R, ribose 5-phosphate; 5C-X, xylulose 5-phosphate; 6C-F, fructose 6-phosphate; 6C-G, glucose 6-phosphate; and 7C, sedoheptulose 7-phosphate. Expression fold changes against the wild-type control at 24 h are presented in green (B) [56].

There are numerous engineered examples of *S. cerevisiae* to enable its xylose utilization [47–55]. Chromosome integration of *YXI*, a synthesized yeast codon-optimized xylose isomerase gene, in *S. cerevisiae* achieved a constitutive expression for the industrial yeast. Use *YXI,* alone with a set of exogenous xylose transporter genes and downstream facilitating genes; several new genotypes were obtained to significantly improved xylose uptake and utilization for cellulosic ethanol production [51, 53, 56]. A signature expression pathway was revealed illustrating interactive relationships of exogenous- and endogenous-source genes for a genetically engineered industrial strain of *S. cerevisiae* [56]. The constitutive expressed *YXI* initiated xylose metabolic pathway and subsequently facilitated interactions with the critical nonoxidative pentose phosphate pathway branch for enhanced xylose uptake and utilization [53, 56] (Figure 3). This strategy can be used as a model to enable NRRL Y-50464 utilizing xylose in the future. ARS developed strain NRRL Y-50464, which is available to the research and development community for continued improvement toward a low-cost cellulosic ethanol production. With the capability to utilize both C-5 and C-6 biomass sugars, cellulosic ethanol productivity of NRRL Y-50464 can be expected to improve significantly.

Currently, the genome sequence of NRRL Y-50464 is not available yet and limited genetic background is known. Genomic study on this strain will reveal fundamental mechanisms of its dual functions aiding its extended improvement and applications. Characterization of the genetic background of NRRL Y-50464, especially for those *β*-glucosidase and related genes, not only benefits the improvement of NRRL Y-50464, but also can be applied to other yeast for strain enhancement and development. With rapid advances of science and biotechnology such as genome editing and CRISPR tools, it is expected that more desirable strains can be accomplished in the future. NRRL Y-50464 can serve a valuable new genetic resource in this regard.

Further process engineering is also needed to improve the conversion efficiency of the bioreactor. Traditional bioreactor is designed for liquid fermentation with bottom stirring, which is not ideal for ethanol production from high solids of pretreated lignocellulosic feedstock. Bottom stirring limits conversion efficiency significantly when solids loading levels are greater than 15% [21]. High solids are required to achieve commercially realistic ethanol titers for distillation efficiency and minimizing process water usage. It is necessary to improve mixing at high solids by altering the stirring mechanism. As observed using a 5-L bioreactor equipped with a helical stirring apparatus, a higher ethanol titer of 38.1 g/L was achieved from 25% solids loading of conventional corn stover with a significant low level of cellulase input of 5 mg protein/g glucan or 6.7 FPU/g glucan [20]. Its conversion efficiency was 55.5%, which is much improved compared to the 32.4% observed in a conventional 2-L bioreactor with a bottom stirring mechanism (Table 1). The helical blade stirring provides more efficient mass transfer of slurry throughout the entire vessel at moderate power input during the SSF process. This suggests the significant importance of a proper bioreactor design to improve the fermentation performance. Better mixing will also lead to better hydrolysis and incur lower shear-related losses in enzyme activities.

Lignin has been well known as a major factor limiting enzymatic biomass deconstruction and higher titers of cellulosic ethanol production, and it was also observed from corn stover conversion using NRRL Y-50464 [21, 57]. Mild-alkali- and natural deep eutectic solvent-pretreated rice straw resulted in significantly higher ethanol production compared to dilute-acid-pretreated rice straw, which was due to the lignin removal by these methods [24, 25]. A significant amount of glucan was also observed to be unavailable for ethanol conversion in corncob residues [36]. More efficient pretreatment methods to remove the inhibitory lignin and release more insoluble biomass sugars are critically needed for an economic downstream yeast fermentation process.

Thus, an integrated improvement is needed, which consists of strain performance, enzyme hydrolytic efficiency, more efficient pretreatment methods, and proper process engineering, to achieve a low-cost cellulosic ethanol production from lignocellulosic materials.[22, 58, 59].

## Figures and Tables

**Table 1:** A survey of cellulosic ethanol production using *β*-glucosidase-producing strains without the addition of extra *β*-glucosidase.

Strain	Substrate	Fermentation apparatus	Solids loading (%)	Cellulase dosage	Time (h)	Ethanol titer (g/L)	Reference
*S. cerevisiae* NAN-227	Corncobs	Flask	7	^*b*^20 IU/g solids	72	20	Shen et al. [14]
*S. cerevisiae* D56	Avicel	Flask	^*a*^8	^*c*^25 FPU/g cellulose	96	26.37	Lee et al. [13]
*S. cerevisiae* INVSc1	Japanese cedar	Tube	10	^*d*^15 mg protein/g solids	72	12	Treebupachatsakul et al. [22]
*S. cerevisiae* SyBE001603	Avicel	Flask	^*a*^4	^*e*^10 FPU/g glucan	144	15.8	Hu et al. [23]
*Clavispora* NRRL Y-50464	Corncob residue	Bottle	20	^*c*^0.15 ml/g solids	72	22	Liu et al. [16]
*Clavispora* NRRL Y-50464	Corncob residue	Bottle	20	^*c*^0.15 ml/g solids	120	26.6	Liu et al. [16]
*Clavispora* NRRL Y-50464	Corncob residue	Bioreactor	25	^*c*^0.2 ml/g solids	120	23	Liu et al. [16]
*Clavispora* NRRL Y-50464	Avicel	Bottle	^*a*^14	^*c*^15.3 FPU/g cellulose	72	40.44	Liu and Cotta [21]
*Clavispora* NRRL Y-50464	Avicel	Bottle	^*a*^14	^*c*^15.3 FPU/g cellulose	120	47	Liu and Cotta [21]
*Clavispora* NRRL Y-50464	SigmaCell	Bottle	^*a*^14	^*c*^15.3 FPU/g cellulose	72	39.64	Liu and Cotta [21]
*Clavispora* NRRL Y-50464	SigmaCell	Bottle	^*a*^14	^*c*^15.3 FPU/g cellulose	120	48	Liu and Cotta [21]
*Clavispora* NRRL Y-50464	Standard NREL corn stover	Bottle	25	^*c*^15.3 FPU/g cellulose	72	17.2	Liu and Cotta [21]
*Clavispora* NRRL Y-50464	Standard NREL corn stover	Bottle	25	^*c*^15.3 FPU/g cellulose	120	23	Liu and Cotta [21]
*Clavispora* NRRL Y-50464	Delignified NREL corn stover	Bottle	25	^*c*^15.3 FPU/g cellulose	72	28.2	Liu and Cotta [21]
*Clavispora* NRRL Y-50464	Standard NREL corn stover	Bioreactor/Conventional	20	^*c*^15.3 FPU/g cellulose	48	32	Liu and Cotta [21]
*Clavispora* NRRL Y-50464	Standard NREL corn stover	Bioreactor/Conventional	20	^*c*^15.3 FPU/g cellulose	120	34.7	Liu and Cotta [21]
*Clavispora* NRRL Y-50464	Rice straw	Bottle	15	^*c*^9 FPU/g solids	36	25	Chapla et al. [24]
*Clavispora* NRRL Y-50464	Rice straw	Reactor/Bottle	20	12 FPU cellic CTec2	24	19.7	Kumar et al. [25]
*Clavispora* NRRL Y-50464	Rice straw	Reactor/Bottle	20	12 FPU cellic CTec2	36	36.7	Kumar et al. [26]
*Clavispora* NRRL Y-50464	Conventional corn stover	Bioreactor/Helical stirring	25	^*f*^5 mg protein/g glucan (6.7 FPU/g glucan)	72	35	Geberedikan et al. [20]
*Clavispora* NRRL Y-50464	Conventional corn stover	Bioreactor/Helical stirring	25	^*f*^5 mg protein/g glucan (6.7 FPU/g glucan)	96	38.1	Geberedikan et al. [20]

^*a*^Commercially available pure cellulose. ^*b*^Cellulase JU-A10 containing 4.3 IU/mL of filter paper activity and 0.8 IU/mL of *β*-glucosidase. ^*c*^Cellulase Celluclast 1.5 L. ^*d*^Cellulase from *Trichoderma reesei* culture supernatant. ^*e*^Cellulase Celluclasta. ^*f*^Cellulase Youtell #7.

**Table 2:** Protein characterization of partially purified *β*-glucosidases from *Clavispora* NRRL Y-50464 and their optimal pH and temperature as measured by specific enzyme activity.

Protein	Amino acid	Molecular WT (Kda)	*p*H	Temperature (˚C)
BGL1	844	93.3	6	45
BGL2	804	88.3	4 and 5	50
BGL3	837	92.5	5	55

**Table 3:** Kinetic parameters of partially purified *β*-glucosidases from *Clavispora* NRRL Y-50464.

Protein	*Vmax* (*μ*mol min^−1^ mg^−1^)	*Kcat* (min-1)	*Kcat/Km* (mM^−^1 min^−1^)	*Km* (mM)	*K i* (mM) (against glucose)	Reference
BGL1	5.91	Na	Na	0.355	15.2	Liu et al. [17]
BGL2	5.27 ± 0.11	547.89 ± 15.91	6,834.23 ± 94.47	0.08 ± 0.01	61.97 ± 2.49	Wang et al. [41]
BGL3	1.63 ± 0.04	84.04 ± 1.06	462.50 ± 14.12	0.18 ± 0.02	38.33 ± 1.15	Wang et al. [41]
Novo188	4.2	Na	Na	0.448	0.735	Liu et al. [17]
